# The Influence of the Menstrual Phases on Polysomnography

**DOI:** 10.7759/cureus.871

**Published:** 2016-11-09

**Authors:** Andrew R Spector, Daniel Loriaux, Diana Alexandru, Sanford H Auerbach

**Affiliations:** 1 Department of Neurology, Duke University Medical Center, Durham, NC; 2 School of Medicine, Duke University Medical Center, Durham, NC; 3 Pulmonary Disease, Critical Care Medicine, Elliot Health System; 4 Neurology, Boston University School of Medicine

**Keywords:** menstrual cycle, obstructive sleep apnea, polysomnography

## Abstract

Purpose: The primary objective of this study is to determine how the phases of the menstrual cycle influence the results of polysomnography (PSG).

Methods: Twenty-eight adult subjects who reported regular menstrual periods, last menstrual period (LMP) within 26 days of their PSG, no exogenous hormone use, no history of polycystic ovarian syndrome, and who were scheduled for diagnostic PSG at Boston Medical satisfied inclusion criteria for the study. These subjects were divided into a Follicular Cohort (days 0-13 of the cycle) or Luteal Cohort (days 14-26 of the cycle), and a one-way analysis using a t-test was performed to test the hypothesis that the follicular phase confers protection against obstructive sleep apnea (OSA). A likelihood-ratio chi-square test was also applied to assess for a statistically significant association between menstrual stage and the presence of moderate-to-severe sleep apnea (apnea-hypopnea index (AHI) > 15/h). Thus, the statistical analysis was performed using AHI as both a continuous and a categorical outcome.

Results: The mean AHI for patients in the Follicular Cohort (6.1/h) was significantly lower than the Luteal Cohort (14.3/h, p = 0.033). In the Follicular Cohort, 12% of patients had moderate to severe OSA. In the Luteal Cohort, 46% of patients had moderate to severe OSA (p = 0.045).

Conclusions: Subjects undergoing PSG during the follicular phase have significantly lower AHIs than those in the luteal phase. Thus, the timing of PSG acquisition for regularly menstruating women should be considered when interpreting results.

## Introduction

Female sex hormones, including estrogen and progesterone, are thought to have a protective effect against obstructive sleep apnea (OSA), which manifests as decreased rates of OSA in regularly menstruating (“premenopausal”) women compared to postmenopausal women or men [1].

The physiologic rationale for the pro-respiratory function of estrogen and progesterone in OSA is multifactorial: (1) both hormones increase pharyngeal dilator muscle activity, thereby resisting a collapse of the upper airway during sleep, (2) both hormones promote hypoxic and hypercapnic ventilatory responses, (3) estrogen inhibits overexpression of hypoxia-inducible factor-1 (HIF-1), a transcription factor that is responsible for decreasing resistance to fatigability in the genioglossus muscle, and (4) progesterone acts on the nuclear progesterone receptor (nPR) transcription factor to augment the ventilatory response to hypoxia [2-5].

Although estrogen and progesterone share redundancy of function as activators of the pharyngeal dilator muscles and stimulators of the respiratory centers, prior clinical research on estrogen and progesterone therapy suggests that there are distinct differences in the therapeutic efficacy for each hormone [6-10]. No study of progesterone monotherapy has demonstrated a therapeutic benefit for obstructive sleep apnea (Table 1). Furthermore, there is evidence that progesterone supplementation in combination with estrogen therapy may attenuate the respiratory benefits of estrogen alone [6].

**Table 1:** Prior Investigations of the Therapeutic Benefit for HRT in OSA AHI: apnea-hypopnea index; HRT: hormone replacement therapy; MPA: medroxyprogesterone acetate; MPG: medroxyprogesterone; OSA: obstructive sleep apnea; PSG: polysomnography

Study	Cohort	Supplement	Study Design	PSG Findings	Conclusions
Keefe, et al., 1999 [11]	5 postmenopausal women	Estrogen & Combination	17-beta-estradiol (E2) for 3-4 weeks followed by E2 + medroxyprogesterone acetate (MPA) for 10-12 days.	E2 or E2+P both had therapeutic benefit in all patients.	Estrogen supplementation or combined therapy has therapeutic benefit in the treatment of OSA. Progesterone monotherapy, however, does not alleviate sleep apnea severity in menopausal women.
Block, et al., 1981 [12]	21 postmenopausal women	Progesterone	11 women received 30 mg medroxyprogesterone (MPG) daily. 10 women received placebo control in a randomized, double-blind control study.	Total AHI prior to MPG therapy was 7.6 (+/- 12.7) and 5.7 (+/- 15) post-MPG therapy. The number of recorded apneas increased from 4.4 (+/-9) pre-MPG to 5 (+/-14) post-MPG.	There is no therapeutic benefit of medroxyprogesterone monotherapy for OSA.
Pickett, et al., 1989 [13]	9 postmenopausal women	Combination	Nine healthy, non-obese postmenopausal women received placebo or combined MPA (20 mg TID) and estrogen (conjugated equine estrogens, Premarin, 1.25 mg BID) therapy for 1-week duration.	Combined therapy decreased the total number of sleep-disordered breathing episodes from 137/night to 28/night.	Combined exogenous estrogen and progestin therapy reduced the number of sleep-disordered breathing episodes in healthy, non-obese, postmenopausal women.
Manber, et al., 2003 [6]	6 postmenopausal women	Estrogen & Combination	All subjects underwent 4 sequential PSG analyses: (1) after no HRT, (2) after two nights on HRT, (3) after 7-12 days on estrogen + placebo, and (4) after 7-13 days of estrogen + progesterone.	Estrogen monotherapy was associated with significant reduction in Total AHI (22.7/hour to 12.2/hour). In contrast, there was no significant change observed for patients on estradiol + progesterone HRT.	Estrogen has a substantial benefit on OSA in postmenopausal women, but these beneficial effects of estrogen HRT are attenuated when progesterone is included.
Cistulli, et al., 1994 [14]	10 postmenopausal women	Estrogen & Combination	The therapeutic efficacy of short-term hormone replacement therapy was studied with either estrogen alone or in combination with progesterone supplementation for 50 days.	Neither estrogen nor combined therapy with estrogen and progesterone achieved a significant reduction in AHI. Although no change in hypercapnic ventilatory responsiveness was observed, there was an increase in hypoxic ventilatory responsiveness.	Short-term HRT is ineffective in the clinical management of postmenopausal women with OSA.
Polo-Kantola, et al., 2003 [15]	62 postmenopausal women	Estrogen	Prospective randomized placebo-controlled double-blind crossover study.	Estrogen replacement therapy decreased the occurrence (p=0.047) and frequency (p=0.049) of sleep apnea.	Unopposed estrogen replacement therapy has a minor therapeutic effect on OSA.
Strohl, et al., 1981 [10]	9 adult patients: 8 Men 1 Woman	Progesterone	PSGs were acquired for all patients in the study, both at baseline and 1-6 weeks following MPA monotherapy (60-120 mg daily).	MPA therapy was associated with significant reduction in PaCO2 and increase in PaO2. Four of the 9 patients reported resolution of daytime somnolence and disappearance of pedal edema with significant weight reduction in 3 of those 4. For these four patients, a significant reduction in the frequency of obstructive apneas was achieved.	Although MPA monotherapy can have a beneficial therapeutic effect in patients with OSA, this is a heterogeneous patient population and this effect is not achieved in all subjects.
Cook, et al., 1989 [8]	10 Men	Progesterone	All ten patients underwent initial PSG before entering a double-blind crossover study using MPA 150 mg daily or placebo. Treatment was continued for 1-week and then followed by a second polysomnogram. A 3-week washout period separated MPA therapy and placebo trial.	No changes in the frequency of respiratory events, the mean duration of respiratory events, or mean fall in O2 saturation was achieved for patients treated with MPA.	MPA monotherapy does not alter indices of severity in OSA.
Rajagopal, et al., 1986 [7]	13 Men	Progesterone	PSG was performed before and after a 4-week treatment period with MPA (60 mg daily) and once again one week following cessation of treatment.	No significant differences in frequency or severity of apneic episodes were achieved.	MPA monotherapy does not improve disordered breathing during sleep in the non-hypercapnic patient with OSA.
Franklin, et al., 1991 [9]	1 premenopausal woman	Estrogen & Combination	Patient refused CPAP therapy and was prescribed combination estradiol (2 mg) daily with MPG (5 mg) daily for the first 10 days of every other month. When medication therapy was discontinued, symptoms returned. Re-initiation of combination therapy once again achieved symptom resolution.	Repeat PSG following 1-year of therapy (during estrogen monotherapy) confirmed improvement in OSA.	Estrogen monotherapy (daily) alternating with combined therapy (first 10 days, every other month) led to complete OSA resolution in a 45 y/o female patient.
Wesstrom, et al., 2005 [16]	4 postmenopausal women, 1 perimenopausal woman.	Combination	Patients received baseline PSG followed by combination HRT (2 mg estradiol, 0.5 mg trimegestone) orally for 5-6 weeks. Repeat PSG was performed to assess therapeutic effect of combination HRT.	Mean Total AHI was used as the outcome measure for the study. The mean Total AHI prior to 5-6 week treatment phase with combination HRT was 14.9. Following treatment, the mean Total AHI across all subjects had been reduced to 3.9. This represents a 75% reduction.	Combination HRT offers an effective alternative therapy for patients affected by OSA.
Bixler, et al., 2001 [1]	1,741 Subjects: 1,000 Women 741 Men	Combination	Cross-sectional, observation study in which 1,741 subjects underwent PSG and sleep apnea severity in HRT patients was contrasted against nonusers.	OSA prevalence in premenopausal women: 0.6%. OSA prevalence in postmenopausal women on HRT: 0.5%. OSA prevalence in postmenopausal women without HRT: 2.7%. OSA prevalence in men: 3.9%	Combination HRT reduces the risk of sleep apnea that is associated with menopause.
Shahar, et al., 2003 [17]	2,852 Women (Age > 50)	Combination	This was an observational study in which AHI was measured for all patients via a single-night, home sleep study. OSA was defined in the study as having an AHI greater than or equal to 15. The prevalence of OSA in women receiving HRT was contrasted against non-users.	OSA prevalence in HRT users: 6.72%. OSA prevalence in non-users: 14.70%.	Combination HRT could have a therapeutic role in the alleviation of sleep apnea.

Only one study, a 62-patient prospective randomized crossover study conducted by Polo-Kantola, et al., investigated short-term unopposed estrogen replacement monotherapy for menopausal OSA patients [15]. Notably, the Polo-Kantola study demonstrated a detectable therapeutic benefit of estrogen monotherapy with decreased occurrence and frequency of sleep apnea [15]. The analogous natural state of elevated estrogen in premenopausal women occurs during the follicular phase of the menstrual cycle. Therefore, rising follicular estrogen as patients approach mid-cycle ovulation could similarly translate into reduced frequency and severity of sleep-disordered breathing on PSG. We hypothesized that premenopausal women would demonstrate lower sleep apnea severity during the follicular phase of the menstrual cycle, the time of highest unopposed estrogen.

## Materials and methods

### Procedure

Permission for this study was obtained from the Boston University Institutional Review Board. Informed consent was obtained from all individual participants included in the study. This study consisted of a chart review of all female patients who underwent polysomnography at the Boston Medical Center Sleep Disorders Center between September 15, 2012 and March 15, 2013 who could recall the first day of their last menstrual period (LMP) and were not using exogenous sex hormones. In order to be included, subjects had to report an LMP of no more than 26 days prior to the study to avoid the potential confounder of early pregnancy. Subjects were excluded if they reported “irregular” menstrual periods or a history of polycystic ovarian syndrome (PCOS). All women included in the study were referred for PSG to evaluate for obstructive sleep apnea. Women referred for PSG with a multiple sleep latency test (MSLT) were excluded.

As eligible subjects were identified, their polysomnogram reports were reviewed to determine the overall apnea-hypopnea index (AHI), rapid eye movement (REM) AHI, and non-rapid eye movement (NREM) AHI. The subjects’ pre-study questionnaires were reviewed to obtain additional baseline characteristics, such as body mass index, race, and recent alcohol use. Subjects were then grouped by LMP. Subjects who reported their LMP within 0-13 days from the date of their PSG were placed in the “Follicular Cohort” while subjects with an LMP 14-26 days prior to their PSG were placed in the "Luteal Cohort". 

### Polysomnography

The digital PSG consisted of the simultaneous recording of electroencephalogram, electrooculogram, electromyogram, electrocardiogram, respiratory effort, thermistor respiratory flow, nasal pressure, pulse oximetry, leg movement, body position, sound, video, and positive airway pressure device. The Viasys Somnostar version 9-1b recording equipment (SensorMedics, Yorba Linda, CA) was used. 

The AHI was calculated as the total number of apneas, plus hypopneas, per hour with hypopneas defined as a flow reduction with a 3% oxygen desaturation or an arousal.   

### Questionnaires

Prior to undergoing PSG, all patients completed a medical history questionnaire. Questions include assessments of daytime sleepiness, medical history (including menstrual history), prescription and illicit drug use, alcohol use on the day of the test, caffeine use, and typical sleep habits. Additional screening questions consisted of last reported menstrual period as well as any past medical history of irregular menses or PCOS.  

### Statistical analysis

AHI was evaluated both as a continuous dependent variable and as a categorical dependent variable with a moderate-to-severe OSA cutoff (AHI > 15/h). A one-way analysis was performed using a t-test to compare the mean overall AHI in the Follicular Cohort with the Luteal Cohort. This analysis was repeated for REM AHI and NREM AHI. A likelihood-ratio chi-square test was applied to assess for a statistically significant association between the stage of the menstrual cycle and AHI > 15/h. 

## Results

Twenty-eight subjects satisfied inclusion criteria for the study. The Follicular Cohort was comprised of 17 women and the Luteal Cohort consisted of 11 women. In the Follicular Cohort, there was one woman who self-identified as White, eight as Hispanic, six as Black, and two who did not report a race. The Luteal Cohort was comprised of two Whites, five Hispanics, one Black, one Indian, and two who did not report a race. None of the women reported drinking any alcohol on the day of the study.  

The mean age for all women was 39.3 years (range: 28 - 51). The mean age in the Follicular Cohort was 37.9 years (range: 28 - 50) compared to 42.3 years (range: 29 - 51) in the Luteal Cohort (p = 0.115) (Table 2). Age was assessed as a potential confounding variable within the dataset and confirmed to have no statistically significant influence on the reported results (p = 0.388).

**Table 2:** Summary of Cohort Demographics BMI: body mass index

Cohort	Total Subjects	BMI (kg/m^2^)	Age (Years)	Age Range (Years)	Epworth
Follicular	17	33.9	37.9	28-51	9.7
Luteal	11	31.8	42.3	29-51	8.1

The mean Epworth Sleepiness Scale score for all women was 9.5. In the Follicular Cohort, the mean was 9.7 (range: 0 - 17). In the Luteal Cohort, the mean was 8.1 (range: 0 - 22, p = 0.08) (Table 2).  

The mean body mass index (BMI) for all women was 33.7 kg/m2. In the Follicular Cohort, the mean was 33.9 kg/m2 (range: 21.7 - 50.7). In the Luteal Cohort, the mean was 31.8 kg/m2 (range: 23 - 41.8, p = 0.40) (Table 2). 

**Figure 1:**
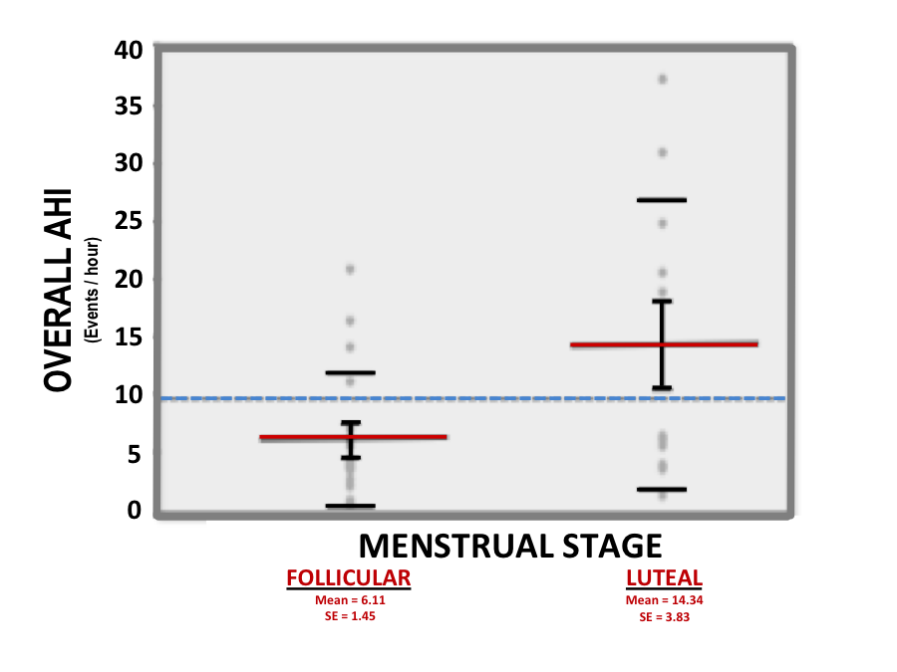
Mean Overall AHI in Follicular Cohort (Left) vs. Luteal Cohort (Right). A one-way t-test was used to evaluate whether reduced Total AHI (apnea-hypopnea index) shares a statistically significant correlation with escalating estrogen during the follicular phase of the menstrual cycle. This one-way analysis confirms a statistically significant difference between the mean Total AHIs in the follicular versus luteal groups (p=0.033). Horizontal solid bars represent the mean Total AHI within each group. The error bars and standard deviations are shown for each mean. The horizontal dashed line represents the mean Total AHI of all subjects.

The mean overall AHI in the Follicular Cohort (6.1/h) was significantly lower than in the Luteal Cohort (14.3/h) (p = 0.033, Figure 1). The mean REM AHI in the Follicular Cohort was 15.1/h versus 30.9/h in the Luteal group (p = 0.099). The mean NREM AHI was 3.6/h in the Follicular Cohort versus 7.8/h in the Luteal Cohort (p = 0.108). The mean supine AHI in the Follicular Cohort was 10.2/h versus 20.6/h in the Luteal Cohort (p = 0.09). The mean non-supine AHI in the Follicular Cohort was 3.1/h versus 10.0/h in the Luteal Cohort (p = 0.09). Of the women who underwent PSG during the follicular phase, 12% had an overall AHI greater than 15/h. In the Luteal Cohort, 46% of patients had an overall AHI greater than 15/h (p = 0.045) (Table 3). 

**Table 3:** Summary of Polysomnography Results AHI: apnea-hypopnea index; NREM: non-rapid eye movement; REM: rapid eye movement

Cohort	n	AHI > 15	Overall AHI	REM AHI	NREM AHI	Supine AHI	Non-Supine AHI
Follicular	17	12%*	6.1*	15.1	3.6	10.2	3.1
Luteal	11	46%*	14.3*	30.9	7.8	20.6	10.0

## Discussion

The epidemiology of obstructive sleep apnea (OSA) exhibits a clear gender disparity; the prevalence of OSA in men (3.9%) is three times the prevalence in women (1.2%) [18-20]. This male predominance of OSA disappears after the age of 50, at which time women show approximately equal prevalence to men [1]. These findings suggest that female sex hormones play a pivotal role in governing patient susceptibility to OSA, which is further supported by research showing significantly decreased levels of estrogen and progesterone in patients who have an AHI greater than 10/hour [21]. Despite this convincing evidence for a strong link between female sex hormones and obstructive sleep apnea, the influence of the menstrual cycle on PSG results has remained largely undefined.

Prior studies investigating the hormonal influence on OSA have shown conflicting results. For example, an 11-patient prospective crossover study by Driver, et al. concluded that the luteal phase of the menstrual cycle was associated with reduced upper airway resistance in premenopausal women [22]. A second 11-patient study by Stahl, et al. showed that none of the PSG sleep or breathing parameters were significantly affected by oscillating progesterone levels during the menstrual cycle [23]. Other research has found that neither the follicular nor the luteal phase altered the rate, duration, or extent of the desaturation associated with sleep disordered breathing during NREM sleep, but that the luteal phase was associated with marginal improvements in these parameters during REM sleep [24]. It is important to recognize, however, that these prior studies were conducted using normal subjects or heterogeneous sample populations incorporating both healthy and symptomatic subjects. None of the women included in the Driver, et al. study, for example, presented with symptoms concerning for OSA and only three women were ultimately found to have an AHI of greater than 10/hour [22]. We also had a larger sample size.

Our study has unique clinical relevance because it is comprised of undiagnosed, premenopausal women referred for diagnostic PSG due to high clinical concern for OSA. In this population of high-risk patients, obtaining accurate PSG results is particularly critical for optimizing the subsequent therapeutic intervention. Our results suggest that the menstrual phases have direct clinical consequences in the diagnosis of OSA; significantly higher overall AHI values were observed in women who underwent PSG during the luteal phase, and women in the luteal phase were nearly four times more likely to return a result of moderate to severe sleep apnea (Figure 1). Although NREM and REM AHI values did not reach statistical significance, this was likely due to limited sample size, and it is important to note that the statistical trends observed for all measures of AHI across the Follicular and Luteal Cohorts were consistent. These findings suggest that PSG results are partially a function of the menstrual phase.

There are several study limitations. First, our sole marker of menstrual phase was patient-reported last menstrual period. Although less reliable than hormonal assays, we applied gynecologic history and patient-recalled LMP in women with menstrual regularity to predict their menstrual phase. We opted to pursue this method because it is a process that would be easy to replicate in clinical settings. Performing hormonal assays on all patients referred for diagnostic PSG, by contrast, would be impractical. Not only is patient-recalled LMP easily incorporable into the pre-PSG patient assessment, but it has also proven to be highly reliable with 81% of women correctly reporting their LMP to within two days [25]. Other study limitations include the limited number of subjects and retrospective study design.

In concordance with prior research that has shown a therapeutic benefit of short-term estrogen monotherapy, our findings suggest that the follicular phase of the menstrual cycle may induce a similarly protective effect against OSA in premenopausal women. With a nearly four-fold increase in moderate to severe OSA in the Luteal Cohort, factoring in menstrual phase could be a clinically significant improvement in obtaining a correct diagnosis and treatment plan for premenopausal women with symptoms of sleep apnea. Our findings could also account for why some women with symptoms of OSA have negative PSGs. Future research should explore the function of continuous positive airway pressure (CPAP) during the menstrual cycle as it is possible that different pressures are required based on these phases. An additional avenue for future research includes performing PSGs on the same women in both phases of the menstrual cycle to assess the magnitude of the AHI difference between phases.

## Conclusions

The results of this study are the first to suggest that the follicular phase of the menstrual cycle has a protective effect against sleep-disordered breathing in a population of premenopausal women who were being referred for diagnostic PSG with high clinical suspicion for OSA. AHI values were significantly higher for patients who underwent PSG during the luteal phase, whereas patients undergoing PSG during the follicular phase demonstrated lower overall REM and NREM AHI values. These findings are clinically relevant because they suggest that premenopausal patients who undergo polysomnography during the first half of their menstrual cycles may present with lower AHI values on PSG, potentially leading to the inaccurate classification of OSA severity and increased morbidity. Consideration of the LMP in the scheduling and interpretation of PSG is encouraged to help minimize these risks. 
